# A scoping review of the effects of serotonergic psychedelics on attitudes towards death

**DOI:** 10.1007/s00213-025-06787-x

**Published:** 2025-04-21

**Authors:** Noah N. T. Barr, Kayla J. Giese, Sam G. Moreton

**Affiliations:** School of Psychology, Faculty of the Arts, Social Sciences and Humanities, University of Wollongong, Northfields Ave, Wollongong, NSW 2522 Australia

**Keywords:** Death Anxiety. Death Attitudes. Psychedelics. Serotonergic Psychedelics

## Abstract

**Rationale:**

Emerging evidence suggests that psychedelic experiences have the potential to change attitudes towards death and reduce death anxiety. Improved attitudes towards death, specifically reduced death anxiety, are of psychological significance for clinical and non-clinical populations alike. Despite this emerging evidence, little is known about the phenomenology of this potential outcome.

**Objectives:**

To provide a systematic overview of studies reporting effects of psychedelics on attitudes towards death and death anxiety, thereby identifying any gaps in the current literature and informing suggestions for future research.

**Methods:**

MEDLINE, Scopus, PsycINFO, and Web of Science were systematically searched for empirical studies that measured attitudes towards death and death anxiety as an outcomes of classical psychedelic use. There were no limits on the date or design of the study.

**Results:**

The thirty-one studies included in the review all reported changes in attitudes towards death and/or changes in death anxiety. Despite finding evidence for psychedelics improving death anxiety, we found significant gaps in the existing research relating to the role of set and setting, potential differences across substances, the underlying psychological mechanisms involved, the potential for worsening of death anxiety, and the role of expectancy and placebo effects.

**Conclusions:**

There is largely consistent evidence that psychedelics can often change attitudes towards death and reduce death anxiety. However, less is known about the reliability and strength of these effects, the conditions under which they are likely to emerge and aspects of the experience that best predict them.

Serotonergic psychedelics are experiencing a significant revival in research interest, with recent studies highlighting their potential to address a range of mental health issues, including anxiety, depression, and substance use disorders (Aday et al. 2020). This resurgence has notably extended to exploring the application of psychedelics in end-of-life care, where they show promise in alleviating distress (Yaden et al. 2022). Preliminary findings also indicate that psychedelics may reduce death anxiety (Moreton et al. 2020). This scoping review aims to synthesize and examine the evolving landscape of research on the influence of psychedelics on death anxiety and attitudes, aiming to provide a clearer understanding of their therapeutic potential in this domain.

## Death anxiety and death attitudes

Death attitudes represent a broad spectrum of beliefs and emotions related to death and the dying process (Lehto and Stein 2009). These attitudes, which range from positive, neutral and negative assessments, significantly influence the development of fears associated with death (Lester et al. 2007). Such attitudes towards death are often deeply impacted by pivotal life events, including near-death experiences or other transformative incidents, which can either challenge or reinforce personal beliefs about life and death (Pehlivanova et al. 2022). Death anxiety can be understood as an affect-laden attitudinal position which reflects the manifest culmination of a variety of death related fears, such as fear of one’s own death or the annihilation of the self, the death of others or the ultimate separation, and guilt and concern at the effect one’s own death will have on others (Lehto and Stein 2009; Zhang et al. 2019; Zuccala et al. 2019; Menzies and Menzies 2023). The terms ‘death anxiety’ and ‘fear of death’ are not always considered synonymous. Yalom (1980) explored potential differentiating factors although opted to use the terms interchangeably, while Wong et al (1994) draw a distinction based on the degree of consciousness and specificity surrounding one’s fear/anxiety towards death. However, herein, we will use these terms synonymously.

The study of death attitudes and death anxiety is important for several reasons. Firstly, death anxiety can lead to profound suffering, especially in those grappling with serious or terminal illnesses, affecting their quality of life and mental peace (Blomstrom et al. 2020). Moreover, a growing corpus of research suggests that death anxiety might indirectly compromise well-being by serving an etiological role in several psychopathological conditions, positioning it as a potential underlying factor that spans across various mental health disorders (Iverach et al. 2014; Menzies and Menzies 2023). This concept has led some researchers to consider death anxiety as a “transdiagnostic construct,” implying it may be involved in the onset of an array of psychological disorders (Iverach et al. 2014; Menzies and Menzies 2023).

The transdiagnostic nature of death anxiety and the etiological role it may play in the development of psychopathology is central to Yalom’s (1980) theory of existential psychodynamics. Yalom (1980, p. 10) posited that “…anxiety is the fuel of psychopathology”, and that whereas classical Freudian psychodynamics hold that conflicts surrounding drive gratification cause the anxiety that gives rise to a variety of psychopathological defence mechanisms, Yalom argued that it was underlying existential concerns that give rise to the anxiety which necessitates the adoption of psychopathological defence mechanisms. Yalom’s position here, as with the broader tradition of existential psychology and psychotherapy, emerged out of European existential philosophy (Yalom 1980; Heidenreich et al. 2021; Chavez-Baldini et al. 2024).

The reality of death has long been recognized within the existential philosophical tradition as being a fundamental element of the human experience that must be confronted and accepted if one is to live a full and authentic life (Belfrage 1975; Heidenreich et al. 2021). Death, as conceptualized within this tradition, is more than a mere universal biological reality—it demands a deeply personal confrontation with one’s own existence that occurs whilst one lives—it is endowed with psychological dimensions beyond biological non-existence (Belfrage 1975; Shim 2020). Confronting and accepting the reality of death presents an opportunity to live a more sincere life wherein individual authenticity can become a possibility (Seto et al. 2016; Shim 2020).

### Psychedelics

Serotonergic psychedelics form a unique category of psychoactive substances, primarily engaging with the serotonergic system by acting as agonists at the serotonin 2A receptor (Nichols 2016). Psychedelics include several well-known compounds, each with distinct pharmacological profiles and effects on consciousness. LSD (lysergic acid diethylamide) and psilocybin (the psychoactive component in “magic mushrooms”) are perhaps the most recognized, alongside mescaline (derived from specific cacti). Other well-known compounds include DPT (N,N-Dipropyltryptamine), DMT (N,N-Dimethyltryptamine), which is the psychoactive ingredient in ayahuasca, and 5-MeO-DMT (5-Methoxy-N,N-Dimethyltryptamine).

Despite their pharmacological differences, these substances share the ability to profoundly alter mood, cognition, and perception, often leading to significant changes in people’s understanding of the world (Nichols 2016). During the acute phase of their effects, these psychedelics can render deeply held beliefs and worldviews more pliable (Carhartt-Harris and Friston 2019), offering a critical period where individuals might re-evaluate and transform their understanding of self, life narratives, and the essence of consciousness (Griffiths et al. 2011; Nayak and Griffiths 2022). This potential for catalyzing profound shifts in perspective and consciousness underscores the therapeutic promise of serotonergic psychedelics, including their ability to facilitate significant personal and psychological transformations.

The immediate subjective experiences induced by serotonergic psychedelics are notably diverse and can range from deeply unifying perceptions and indescribable experiences to a profound sense of meaning and self-transcendent emotions, including awe (Griffiths et al. 2016). Conversely, individuals may encounter challenging emotions, such as fear and anxiety, during these experiences. The factors determining the valance of a psychedelic experience are complex and currently the focus of in-depth research (Aday et al. 2021).

The current revival of psychedelic science has led to numerous clinical trials, many of which are still underway, aimed at investigating how psychedelics might benefit various mental health conditions (Ziff et al. 2022). The preliminary results from these studies are encouraging, suggesting that psychedelics might not only offer symptom relief but also induce lasting changes in psychological structure (Uthaug et al. 2022; Yaden et al. 2016). Importantly, the findings relevant to this review indicate that psychedelics have the capacity to alter deep-seated beliefs about the cosmos, relations to self and other, aid in the acceptance of existential truths, and modify the handling of challenging emotions (Nayak et al. 2023; Moreton et al. 2023a, b; Sweeney et al. 2022), pointing to their profound effect on our understanding of and engagement with the world. Among these existential shifts, psychedelics may influence attitudes toward death through multiple mechanisms (Moreton et al. 2020), including fostering death acceptance, reducing death anxiety, or modulating death avoidance—all of which may have clinical relevance.

### Purpose of the scoping review

Amidst the burgeoning interest in the intersection of psychedelics and mental health, a comprehensive examination of how these substances relate to death attitudes remains absent. While recent literature reviews have delved into the use of psychedelics among individuals facing life-threatening illnesses (Whinkin et al. 2023; Yaden et al. 2022), their scope has been confined to clinical trials, omitting a broader spectrum of research including retrospective analyses, qualitative studies, case reports, and experimental work beyond severe illness contexts. This review seeks to bridge this gap by offering a systematic exploration of the existing body of research on the impact of psychedelics on death anxiety and attitudes towards death. Our aim is to shed light on the following questions:

- Which psychedelics have been studied in relation to death attitudes, and do they exhibit differential effects?
- What approaches have researchers taken to define and measure death anxiety or attitudes in the context of psychedelic studies?
- Is there documentation and analysis of potential increases in death anxiety following psychedelic use?
- Which immediate subjective experiences during psychedelic states have been linked to shifts in death anxiety?
- Are there identified long-term impacts of psychedelic use that influence death anxiety?
- How do the set and setting of psychedelic experiences affect outcomes related to death anxiety?
- Have the influences of participant expectations and study blinding been considered?

By addressing these inquiries, this review aims to comprehensively map the landscape of psychedelics research in relation to death anxiety, identifying areas for future investigation and potential therapeutic applications.

## Method

### Search Protocol

The search protocol for this scoping review was developed in line with PRISMA-ScR guidelines (Page et al. 2021). The databases selected for the literature search were MEDLINE, Scopus, PsycINFO, and Web of Science. The search strategy was peer-reviewed by an information specialist using the Peer Review of Electronic Search Strategies (PRESS; McGowan et al. 2016) checklist before commencing the search. A full list of search terms is available in the Supplementary Materials.

### Inclusion and exclusion criteria

**Fig. 1:**
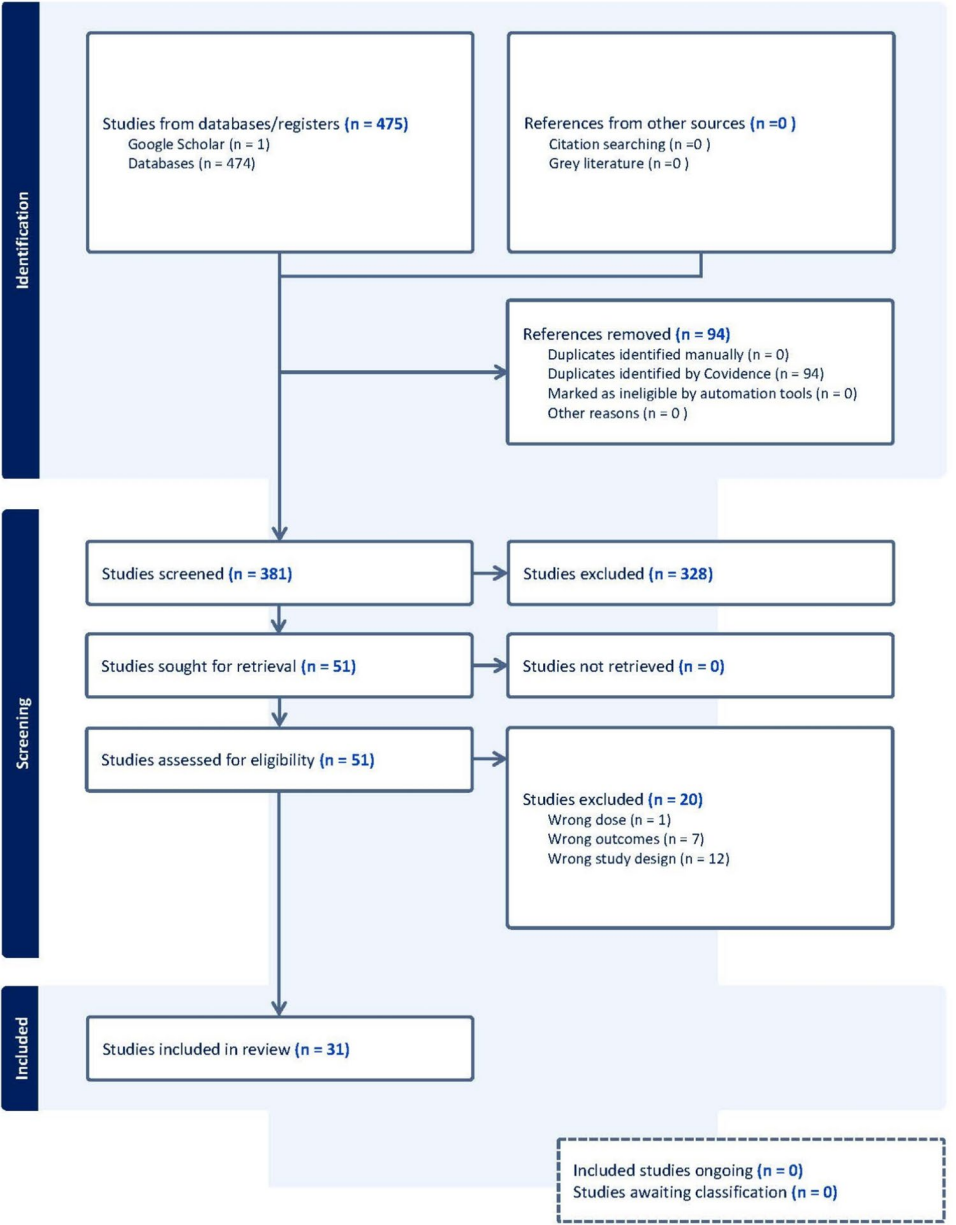
PRISMA Diagram of study identification

The search targeted empirical and peer-reviewed studies regarding the effects of psychedelics on death attitudes and death anxiety. The researchers also manually searched for relevant articles. Studies had to be reported in English and from any location, with human subjects of any age or sample size. Both qualitative and quantitative designs were included, but review papers and theoretical articles were excluded. Psychedelics referred to classical psychedelics including LSD, DPT, Psilocybin, DMT, Ayahuasca, and Mescaline (Nichols 2016), but excluded MDMA and Ketamine. There were no restrictions on the date of publication. As seen in a PRISMA diagram below (Fig. 1), there were 475 articles initially identified. An initial screening excluded 328 articles that did not meet the criteria of being empirical explorations of the effect of psychedelics on death attitudes and death anxiety. After removing duplicates and manually screening to ensure studies fit the inclusion criteria, this was reduced to a final sample of 31 articles.

## Results

This section will present the results of this scoping review as they pertain to the research questions listed above. For a detailed overview of the 31 articles included in this review, see Table 1 below.

**Table 1:** Overview of included articles: key details and main findings

First author, year	Study design, substance investigated & outcome measure	Set & setting	Acute correlate/s with death attitudes/anxiety	Enduring correlate/s with death attitudes/anxiety	Blinding & expectancy	Worsening reported	Main finding relating to change in attitudes towards death
Agin-Liebes et al. 2020	Long term follow up of Ross et al 2016 RCT investigating psilocybin for anxiety & depression in patients with terminal cancer diagnosis which used the Death Anxiety Scale	Hospital setting in conjunction with psychotherapy, set not measured	Correlation not measured	Correlation not measured	Original study double blind – success of participant blinding not measured, guide blinding unsuccessful, and expectancy not measured	No	Enduring reductions in fear of death at 6.5–8 months, 3.2 & 4.5 years post final dose
Agin-Liebes et al. 2021	Cross-sectional study exploring the effect of naturalistic mescaline use on psychiatric improvement and enduring positive life changes which used a Single statement Likert scale to measure death attitudes	Set and setting not measured	Correlation not measured	Correlation not measured	Blinding n/a, expectancy not measured	Combined reports of worsening and no change into one group – e.g. 25/184 participants in depression subgroup reported no change or worsening of attitudes towards death	Improvements in attitudes towards death reported following mescaline use (n = 452 reported an average improvement score of 1.2 on −3 to + 3 scale)
David et al. 2023	Cross-sectional study exploring ayahuasca-induced personal death experiences and their impact on attitudes towards life, death & the environment which used the Death Anxiety Scale, Death Transcendence Scale, and Likert-scale single-item statement to measure death anxiety/attitudes	Ceremonial setting, set not measured	Stronger experiences of ‘ego-dissolution’ (EDI-S) were significantly correlated with higher DTS scores. Occurrence of ayahuasca-induced personal death experience correlated with stronger DTS scores	Changes in attitudes towards death following experience predicted engaged living scores	Blinding n/a, expectancy not measured	No	Significantly stronger endorsement of death transcendent views in those participants who had ayahuasca-induced personal death experience than those who did not have an ayahuasca-induced personal death experience
Davis et al. 2020	Cross-sectional mixed-method survey exploring the phenomenology, interpretations, and effects of entity encounters during DMT experience which used the Entity Survey & content analysis to explore attitudes towards death	Set and setting not measured	No correlation reported	No correlation reported	Blinding n/a, expectancy not measured	No	76% (n = 2561) reported “positive desirable change” in attitudes about death. A content analysis of qualitative data found that 7% of participants reported receiving a message or insight about death
Doblin. 1991	Mixed method long term follow up of Pahnke’s 1962 ‘Good Friday Experiment’ exploring psilocybin & mystical experiences using re-administration of original questionnaire and thematic analysis of interview transcripts in which changes in attitudes towards death were captured	Religious setting with religiously oriented participants, set not measured but Doblin explains “mood was positive and expectant”	Mystical experience qualitatively reported as being associated with reduced death anxiety. Experiences of death/dying described as being associated with reduced fear of death	Qualitative reports of increased political involvement due to feeling more empowered to take risks following reduction in fear of death, as well as changes in metaphysical beliefs which changed attitudes towards death	Original study was double blind – blinding failed, expectancy not measured but positive expectation implied (see set & setting box)	No	Several participants reported experiences which led to changes in their attitudes towards death, their perceptions of what death is, and reductions in their fear of death
Fisher. 1970	Case report of terminally ill patients who underwent LSD-assisted psychotherapy within which changes in attitudes towards death were explored	Supportive hospital setting, set not measured, however authors explain that they ensured that patients had certain psychological attributes including being “… motivated toward psychotherapy” and having “…sufficient ego strength”	Psychological insights, challenging experiences, and peak/mystical type experiences qualitatively reported as correlating with reduced death anxiety	Qualitative report of close association between enduring increases in sense of meaning, increased interpersonal connectedness and reduced death anxiety	Double blind—success not measured, expectancy not measured	No	Three case studies provided which all display improved attitudes, namely reductions in fear of death
Gasser et al. 2015	Mixed method long term follow up of Gasser et al. 2014 RCT investigating LSD for anxiety associated with life-threatening diseases which used qualitative content analysis of semi-structured interviews to capture changes in death attitudes	Supportive setting with two familiar guides, set not measured	Psychological insights and the navigation of challenging experiences qualitatively suggested to be associated with changes in attitudes towards death	Increase in quality of life qualitatively suggested as being caused by an increase in one participant’s improved ability for “…approaching death with equanimity.”	Original study was double blind – blinding unsuccessful, expectancy not measured	No	Seven of nine participants qualitatively reported reductions in fear of death
Gonzalez et al. 2019	Cross-sectional mixed method survey comparing effect of ayahuasca use with peer-support group attendance on grieving process wherein participants were asked whether ayahuasca had a beneficial impact on “…my ability to integrate a transcendental dimension of life and death.”, and further changes in attitudes towards death were captured in a content analysis of the open-ended reports made by participants	Setting and set not measured, however, participants were actively grieving	Psychological insights and navigating challenging experiences qualitatively reported as being associated with changes in attitudes towards death	No correlation reported	Blinding n/a, expectancy not measured	No	76.7% (n = 23) reported beneficial impact on ability “to integrate a transcendental dimension of life and death.” Seven participants qualitatively reported death-related belief changes following the experience e.g. *“that there is life after death.”*
Griffiths et al. 2011	Randomised control trial exploring dose-related psilocybin-induced mystical experiences in healthy participants which used the Death Transcendence Scale to capture changes in death attitudes	Highly supportive setting, set not measured	No correlation reported	No correlation reported	Double blind – success of participant blinding not measured but guide blinding was successful, expectancy not measured	No	Improvements found in specific attitudes towards death – sense of continuation after death increased
Griffiths et al. 2016	Randomised control trial investigating effect of psilocybin on anxiety and depression in participants with life-threatening cancer which used the LAP-R death acceptance subscale & Death Transcendence Scale	“Living room” setting with two familiar guides and efforts were made to allow for a mindset where participants could “trust, let go and be open”, but no measure of set	Mystical experiences (MEQ30) correlated with improvements in both measures of attitudes towards death	No correlation reported	Double blind – success of participant blinding not measured and guide blinding partially successful, expectancy not measured	No	Average scores on the death acceptance measure significantly increased and sustained at 6 month follow-up, as did scores on the Death Transcendence Scale
Griffiths et al. 2018	Randomised control trial exploring the effect of administering psilocybin alongside varying levels of supported spiritual and meditation practices on psychological functioning and prosocial traits which used the LAP-R death acceptance subscale & Death Transcendence Scale	“Aesthetic living room-like environment” with two familiar guides, set not measured	Mystical experience correlated with Death Transcendence	No correlation reported	Double blind – success of participant blinding not measured and guide blinding partially successful, expectancy not measured	No	Greater DTS scores reported for both high-dose groups compared to low-dose group, with high-dose high-support group reporting highest score, LAP-R Death Acceptance scores not reported
Griffiths et al. 2019	Retrospective survey comparing “God encounter experiences” occasioned by LSD, psilocybin, DMT, ayahuasca, and naturally wherein participants were given the option to endorse two statements, either that the experience “Decreased fear of death” or “Increased fear of death”	Set and setting not measured	No correlation reported	No correlation reported	Blinding n/a, expectancy not measured	Yes – 3% of participants endorsed the statement that they experienced an “Increased fear of death.”	Decreased fear of death reported by 70% of participants (n = 3476) as indicated by endorsement of statement that they experienced a “Decreased fear of death”
Grof et al. 1973	Non-randomised experimental study exploring the effect of LSD & DPT on emotional & physical suffering of terminal cancer patients wherein observational data of patient “Fear of death” or “Calm acceptance of death” was collected by therapist, co-therapist, physician, nurse, family member, and independent rater	Highly supportive setting with therapists present, set not measured	The navigation of challenging experiences and peak/mystical experiences qualitatively reported by authors as being associated with improved attitudes towards death	No correlation reported	No blinding attempted, expectancy not measured	Partial report – two patients reported worsened “global index” post-treatment which includes measure of death anxiety	Average fear of death ratings showed significant decrease, however, there was poor inter-rater reliability
Kast et al. 1964	Randomised control trial investigating the analgesic action of LSD in gravely ill patients within which the author described improvement in attitudes towards death in discussion based on observation of trial patients	Hospital setting, participants all experiencing severe pain, set not measured	Peak-like experiences suggested by authors to be closely associated with reductions in fear of death	No correlation reported	No blinding attempted, expectancy not measured	No	Positive impact reported – “these patients displayed a peculiar disregard for the gravity of their situations, and talked freely about their impending death with an affect considered inappropriate in our western civilization, but most beneficial to their own psychic states.”
Kast. 1966	Non-randomised experimental study exploring effect of LSD on the emotional & physical distress of patients with terminal illness wherein attitudes towards death were measured by observation and by daily interviews for three weeks post-dosing	Hospital setting with terminally ill patients given weeks/months to live, set not measured	No correlation reported	No correlation reported	No blinding attempted, expectancy not measured	No	Author reports that a “…change in philosophical and religious approach to dying took place which is not reflected in the numerical data”. Numerical data showed temporary strong reduction in fear of death during active window of experience that returned close to baseline at 3-week follow-up
Kast. 1967	Non-randomised experimental study exploring effect of LSD on the physical & emotional distress of terminally ill patients wherein observational data was collected daily measuring patient “Approach to illness and death” for three weeks post-dosing	Hospital setting wherein patients had terminal diagnosis and were unaware they were receiving LSD, set not measured	No correlation reported	No correlation reported	Single blind (participants were not informed that they were receiving LSD), expectancy not measured	No	Temporary significant improvement – *“Under LSD, patients were so strikingly unconcerned about death or any other anticipatory concern…”.* However, returned close to baseline at 3 week follow-up
Kurland et al. 1972	Non-randomised experimental study and case reports exploring effects of LSD & DPT on the physical & emotional distress in patients with terminal cancer diagnosis wherein ratings were provided by therapist, co-therapist, physician, nurse, family member, and independent rater for patient “Fear of death”	Highly supportive setting with therapists present, set not measured	Mystical experience involving “ego-death and rebirth” qualitatively reported by authors as being causally related with changes in attitudes towards death	No correlation reported	Blinding not attempted, expectancy not measured	Partial report – 8.3% of participants reported worsened “global index” post-treatment which includes “Fear of death”	Average “Fear of death” scores reflect significant decrease, however, poor inter-rater reliability
Lawrence et al. 2022	Naturalistic study using qualitative analysis to explore participant inhaled DMT experiences posted on Reddit, within a portion of which “Accepting death or removed fear of dying” was identified as a theme	Setting reported, set not measured	No correlation reported	No correlation reported	Blinding n/a, expectancy not measured	No	Acceptance of death or reduced fear of death reported following 1.6% of experiences (n = 3778)
Maia et al. 2021	Study of the effect of ritual ayahuasca use on the way individuals understand and relate to their illness, wherein changes in attitudes towards death were captured using thematic analysis of semi-structured interviews	Ceremonial setting, all participants had severe physical illness, set not measured	Ayahuasca-induced introspection allowed for reprocessing of autobiographical content, resulting in improved mental health which authors say was closely associated with reduced fear of death	No correlation reported	Blinding n/a, expectancy not measured	No	Increased acceptance of death and reduced fear of death qualitatively reported
Michael et al. 2023	Naturalistic study of non-clinical DMT use whereby participants were observed during their experience at home and then participated in a semi structured interview which underwent thematic and content analysis, wherein changes in attitudes towards death were identified	Researchers observed participants use DMT in setting of their choice, set not measured	Psychological insights and challenging experiences qualitatively reported as being associated with changes in attitudes towards death	No correlation reported	Blinding n/a, expectancy not measured	No	Qualitative reports of reduced fear of death and improved attitudes towards death
Moreton et al. 2023a, b	Retrospective survey reporting pre/post changes in DAP-R fear of death subscale resulting from a meaningful experience with classical psychedelics	Set and setting not measured	Mystical experience correlated with reduced death anxiety	Enduring increases in subjective well-being correlated with reduced death anxiety	Blinding n/a, expectancy not measured	Yes – 16.92% of participants reported worsened death anxiety	Decrease in death anxiety reported by 79.10% of participants (n = 201) from before (M = 3.67, SD = 1.59) to after (M = 2.69, SD = 1.36) a significant psychedelic experience
Moreton et al. 2023a, b	Retrospective survey reporting pre/post changes in Collett-Lester Fear of Death Scale – Revised resulting from meaningful experience with a classical psychedelics	Set and setting not measured	Mystical experience correlated with reduced death anxiety	Enduring decreases in OCD symptomology correlated with decreased death anxiety	Blinding n/a, expectancy not measured	No	Significant average reductions across all subscales of the CLFDS-R
Pahnke et al. 1969	Non-randomised experimental study exploring the effect of LSD on terminal cancer patients wherein participant fear of death was captured by observational reports made by the participant’s physician, nurse, therapist, and family, and written subjective reports by participants themselves	Highly supportive setting with therapist present, set not measured	Peak experiences reported by authors as being correlated with clinical improvement which includes measure of fear of death	No correlation reported	Blinding not attempted, expectancy not measured	No	14/22 participants scored positively on a “global index” measure comprised of six factors including “fear of death”. Case reports indicate positive change in attitude towards death
Richards et al. 1972	Non-randomised experimental study exploring the effect of LSD on terminal cancer patients wherein participant fear of death was captured by observational reports made by the participant’s physician, nurse, therapist, family, and independent social worker	Highly supportive setting with therapist present, set not measured	Mystical/peak experiences, psychological insights and the navigation of challenging experiences qualitatively reported by authors as being associated with reduced fear of death	Two case studies suggested that improved attitudes towards death resulted in improved interpersonal relations with family members during final stages of life	Blinding not attempted, expectancy not measured	No	Average fear of death scores reflect significant decrease, however, poor inter-rater reliability
Richards et al. 1980	Non-randomised experimental study exploring the effect of DPT on terminal cancer patients which used observational ratings made by two independent raters to capture changes in participant “Denial of the possible imminence of death” & “Fear of death”	Highly supportive setting with therapist present, set not measured	Mystical experience qualitatively reported by authors as being directly connected with reduced death anxiety	No correlation reported	Blinding not attempted, expectancy not measured	No	Decreases in fear of death reported by therapists but not supported by independent raters
Ross et al. 2016	Randomised control trial exploring the effect of psilocybin on anxiety and depression in participants with life-threatening cancer which used the Death Anxiety Scale and Death Transcendence Scale	Supportive setting with therapists present, set not measured	No correlation reported	No correlation reported	Double blind – success of participant blinding not measured and guide blind unsuccessful, expectancy not measured	No	Improved attitudes towards death reflected in increased death transcendence scores but no reductions in death anxiety
Soares et al. 2023	Qualitative investigation of the potential for LSD, DMT, Psilocybin, Ayahuasca, and Mescaline to be used as a means of self-care, wherein a content analysis of semi-structured interviews captured changes in attitudes towards death	Setting measured, set not measured	Out-of-body experience qualitatively reported by one participant as being “one of the most valuable experiences of making peace with death.”	No correlations reported	Blinding n/a, expectancy not measured	No	Qualitative reports of changes in perspectives on death for 6/19 participants
Sweeney et al. 2022	Retrospective survey comparing experiences induced by LSD, DMT, Ayahuasca, Psilocybin to near-dear and other non-ordinary experiences on attitudes towards death which used the DAP-R to capture death attitudes	Set and setting not measured	No correlation reported	No correlation reported	Blinding n/a, expectancy not measured	Yes – 6% reported increased fear of death	89% of participants (n = 2259) endorsed the statement that they experienced “Decreased fear of death” as a result of a psychedelic experience. The mean change score on the Fear of Death subscale of the DAP-R was −2.13 (SD = 1.66)
Swift et al. 2017	Qualitative long-term follow-up of Ross et al (2016) RCT which explored the effect of psilocybin on cancer-related psychological & existential distress, wherein an interpretive phenomenological analysis of semi-structured interview data was used to capture attitudes towards death	Highly supportive setting with therapists present, set not measured	Confrontations with death and death-like experiences, as well as mystical type experiences of interconnectivity qualitatively reported by authors and participants as being associated with reduced death anxiety and improved attitudes towards death	Qualitative report of enduring feelings of connectedness to others and nature resulting in reduced fear of death	Original study was double blind – success of participant blinding not measured and guide blinding unsuccessful, expectancy not measured	No	Enduring improvements in attitudes towards death reported in 11/13 participants (5 participants interviewed within one week of last dose and 8 at 1 year post-dose)
Uthaug et al. 2022	Retrospective survey exploring naturalistic mescaline use wherein attitudes towards death were captured by the Persisting Effects Questionnaire	Setting measured, set not measured	No correlation reported	No correlation reported	Blinding n/a, expectancy not measured	No	Mean change score in attitudes towards death resulting from “most memorable” mescaline experience was 1.2 (SD = 1.2) on a scale from −3 to + 3
Yaden et al. 2016	Retrospective survey comparing psychedelic and non-psychedelic mystical experiences wherein participants were asked to report on a 5-point scale how much the experience “Reduced fear of death”	Set and setting not measured	No correlation reported	No correlation reported	Blinding n/a, expectancy not measured	No	Participants reported the degree to which their psychedelic induced “Religious, mystical, and spiritual experience” influenced their fear of death as being 4.44/5 (n = 331)

### Substances investigated

There were six studies that looked at multiple classical psychedelics (e.g., LSD, DMT, ayahuasca, psilocybin, and mescaline), and two studies that looked at both LSD and DPT. Out of the studies that looked at a specific psychedelic, seven studies investigated the effects of LSD, seven psilocybin, three N,N- DMT, three ayahuasca, one DPT, and two mescaline. Of the six studies that included multiple classical psychedelics, only two reported the comparative effects on death anxiety of each substance. According to Griffiths et al (2019), reductions in death anxiety were reported in 70% of the psilocybin group (n = 1184), 67% of the LSD group (n = 1251), 73% in the ayahuasca group (n = 435), and 77% in the DMT group (n = 606). Sweeney et al (2022) reported that reductions were observed in 91% of the psilocybin group (n = 766), 86% in the LSD group (n = 904), 90% in the ayahuasca group (n = 282), and 90% in the DMT group (n = 307).

### Approaches to measuring death anxiety and death attitudes

There was broad range of approaches taken to measuring death anxiety and death attitudes. In the quantitative portion of articles reviewed the Death Transcendence Scale (Vandecreek and Nye 1993) was used most frequently, totalling five uses. Second to this was the Death Anxiety Scale (Templer 1970), with three uses. Other validated measures such as the Death Attitudes Profile – Revised (Wong et al 1994) and Death Acceptance subscale of the Life Attitude Profile – Revised (Recker and Peacock 1981) were each utilized twice, though most other measures were only used once across the articles reviewed. Several articles used single-item statement measures, for example Griffiths et al (2019) had participants respond with one of three options (decrease, no change, increase) to the question “Did the experience change your fear of death”. Other studies utilized external ratings of participant fear of death, such as in the articles published by Grof et al (1973) and Kurland et al (1972). There were also several qualitative articles that reported changes in attitudes towards death via a variety of coding methods or in the form of case reports. Please see Table 1 for the details of the measured used within each article.

### Potential worsening

Three studies reported psychedelic induced increases in death anxiety; Griffiths et al. (2019) reported that 3% of participants endorsed the statement that their fear of death had increased, Moreton et al (2023a, b) reported a psychedelic-induced increase in death anxiety in 16.92% of participants as measured by the fear of death subscale of the DAP-R, and Sweeney et al (2022) reported that 6% of participants endorsed the statement that their fear of death had increased. The average amount of time between the psychedelic experiences in question and the collection of this data was 8.8 years for Griffiths et al (2019), two years for Moreton et al (2023a, b), and 6.7 years for Sweeney et al (2022).

### Correlates of acute experience

Fourteen articles assessed quantitative measures of acute experience, with five of these studies testing for correlations between acute effects and changes in death anxiety and attitudes towards death. Four articles by Moreton, Arena et al. (2023a), Moreton, Burden-Hill et al (2023b), Griffiths et al (2016), and Griffiths et al (2018) found significant correlations between the psychedelic-induced mystical experiences (as measured by the MEQ30) and altered death attitudes. Two of these studies tested for a correlation between the acute effect of psychological insight and reduced death anxiety, using the Psychological Insight Questionnaire (Davis et al. 2021), with one study reporting no significant correlation (Moreton, Arena et al. 2023a) and the other a correlation that became insignificant when included in a model alongside MEQ-30, which was a more robust predictor (Moreton, Burden-Hill et al. 2023b). David et al. (2023) found a significant correlation between Death Transcendence Scale scores and scores on the Ego Dissolution Inventory (Nour et al. 2016). Additionally, this study reported a significant positive correlation between the intensity of ayahuasca-induced death-like experiences and scores on the Death Transcendence Scale.

Eleven articles made qualitative suggestions that a variety of acute experiential factors—primarily mystical experiences, psychological insights, and the navigation of challenging experiences—were associated with changes in attitudes towards death (Kast and Collins 1964; Fisher 1970; Kurland et al 1972; Richards et al 1972, 1980; Grof et al. 1973; Doblin 1991; Swift et al. 2017; Gonzalez et al. 2019; Maia et al. 2021; Michael et al. 2023). Two particularly salient quotes elucidate such suggestions: *“…subjects who have experienced the ego-death and rebirth in a psychedelic session usually claim that they feel a very radical change in their attitude towards death as a result of this experience. Those who experience feelings of cosmic unity indicate that they are in a state of mind where physical death appears irrelevant*.” (Kurland et al 1972, p. 670), and *“…it remains clinically apparent that the persons who encounter such mystical experiences usually are those who subsequently are most free of anxieties about the process of dying and death itself”.* (Richards et al. 1980, p. 22).

### Correlates of longer term changes

While long term changes—such as increases in feelings of connectedness to others and increases in feelings of purpose and meaning—were commonly reported, only three studies reported quantitative correlations between changes in death attitudes and other long-term changes. One of the studies reported correlations between enduring reductions in death anxiety and increases in subjective well-being (Moreton, Arena et al. 2023a); one reported correlations between enduring reductions in death anxiety and obsessive compulsive disorder symptomology (Moreton, Burden-Hill et al. 2023b); and one reported a correlation between the degree to which participants attitudes toward death had been improved by ayahuasca-induced personal death experience and higher scores on the Engaged Living Scale (David et al. 2023). While several enduring outcomes were reported qualitatively, only four articles directly suggested links between reduced death anxiety and various other long-term effects. Richards et al. (1972) described two cases wherein both participants experienced improved interpersonal relations seemingly due to improved death attitudes; Gasser et al. (2015) reported participant statements which indicate improved attitudes towards death correlated with improved quality of life; Swift et al. (2017) described the relationship between enduring feelings of increased connectedness and decreased death anxiety; and Fisher (1970) reported a connection between increased meaning and reduced death anxiety.

### Set and setting

As 17 studies were experimental studies, there was strict control over set and setting. Typically, the type of set and setting that was utilized in these experiments involved several therapy sessions preceding the dosing session, the constant presence of at least one trained guide, the use of specifically selected music played through headphones, the use of an eyeshade, and often attempts were made to ensure the environment was aesthetically pleasing by providing fresh flowers (e.g. Kurland et al. 1972; Grof et al. 1973; Griffiths et al. 2016, 2018). An additional two non-experimental studies (Maia et al. 2021; David et al. 2023) required that participants undergo their ayahuasca experience in a ritual/ceremonial setting. Four naturalistic studies reported on various aspects of set and setting such as whether experience took place indoors or outdoors, if music was played, and if a guide was present (Lawrence et al. 2022; Michael et al. 2023; Uthaug et al. 2022; Soares et al. 2023). However, none of the studies reported on relationships between set and setting and changes in death attitudes or death anxiety.

### Expectancy and blinding effects

Five studies were double-blind, four of which reported on the degree of success in blinding of the study guides (Griffiths et al. 2011, 2016, 2018; Ross et al. 2016), and one did not report guide blinding success (Fisher 1970). These five studies either did not measure or did not report whether participant blinding was successful. An additional two articles– both long-term follow-ups, one of Pahnke’s 1962 ‘Good Friday Experiment’ (Doblin 1991) and the other of Gasser et al. (2014) (Gasser et al 2015), reported that double blinding was unsuccessful in the original studies. No studies measured expectancy specific to death attitudes at baseline and thus it remains unknown whether expectation affected changes in attitudes towards death.

### Summary of the evidence base

While it is outside the purview of a scoping review to assess the strength of the evidence contained within each article, there are several indicators that psychedelic induced improvements in attitudes towards death may be reliable and enduring, and independent of treatment model or demographic factors. Regarding the reliability of this outcome, this review identified numerous reports across a broad spectrum of contexts wherein positive changes in attitudes towards death were observed in significant portions of the sample. As for how enduring these changes are, positive changes were found to be sustained at six months post-dosing by Griffiths et al (2016) in a randomized double-blind study, and at 6.5–8, 3.2, and 4.5 years in a long-term follow-up of a randomized control trial by Agin-Liebes et al (2020). Positive attitudinal changes were reported in both naturalistic (e.g. Agin-Liebes et al 2021) and controlled hospital settings (e.g. Kurland et al 1972), in samples with terminal illnesses (e.g. Fisher 1970) and good health (e.g. Griffiths et al 2018). However, there remain several unanswered yet critical questions, such as the role of dose–response effects, expectancy effects, dosing schedule effects, and inter-substance effect variance.

## Discussion

The body of literature examined in this review spans a wide array of methodologies, participant demographics, clinical and naturalistic environments, as well as a variety of classical serotonergic psychedelics. The overarching evidence supports the claim that psychedelics may indeed possess the potential to improve attitudes towards death and mitigate death anxiety. The following discussion synthesizes key findings and illuminates critical avenues for future research.

### The role of substance, acute and enduring effects, and set and setting on attitudes towards death and death anxiety

There were seven studies wherein multiple substances were included, but only two reported the comparative outcomes for each specific drug (Griffiths et al. 2019; Sweeney et al. 2022). While the results of the two comparative studies indicated that psychedelic-induced changes in attitudes towards death occurred regardless of which classical psychedelic was used, variation in substance-specific death attitudinal change has only been studied as a point of peripheral interest. Griffiths et al. (2019) found that 77% of participants in the DMT group reported decreased fear of death, while 67% of participants in the LSD group reported a decrease. Griffiths et al (2019) likewise reported stronger mystical experiences in the DMT group (73%) than the LSD group (61%). Sweeney et al (2022), however, reported minimal difference in decreases in fear of death across the same spectrum of substances as Griffiths et al. (2019). As the Sweeney et al. (2022) article was specifically focused on psychedelic-induced changes in attitudes towards death, the possibility of a selection effect must be considered as participants were those who had experienced a psychedelic-induced change in attitude towards death, thereby obscuring any real inter-substance differences. This underscores the need for further research to delineate the distinct effects of various psychedelics on death anxiety. However, given the potential link between the strength of the mystical experience and reductions in death anxiety, it may be hypothesised that the serotonergic psychedelics that induce stronger mystical experiences also typically show greater reductions in death anxiety.

Research into the mechanisms through which the acute elements of psychedelic experiences influence attitudes towards death remains limited. Earlier investigations highlighted the significance of ‘peak’ experiences, characterized by profound awe, a sense of timelessness, and transcendence, bearing resemblance to mystical experiences (Cummins and Lyke 2013; Mosurinjohn et al. 2023). These experiences were believed to be key in facilitating the therapeutic benefits seen in psychedelic use—a notion supported by multiple studies reviewed here. Recent studies employing the Mystical Experience Questionnaire (MEQ) have found mystical experiences to be consistently associated with improved attitudes towards death (Griffiths et al. 2016, 2018; Moreton, Arena et al. 2023a).

However, the relationship between other acute elements and changes in death attitudes is not as clear. For instance, contrary to their hypotheses, investigations by Moreton, Arena et al. (2023a) and Moreton, Burden-Hill et al. (2023b) found that the Psychological Insights Questionnaire was not a robust predictor of changes in death anxiety. It is also conceivable that challenging experiences, especially those entailing a direct encounter with death, may be instrumental in altering death attitudes, a la Grof (2008). Moreton et al. (2020) theorized about such dynamics, and this was empirically explored by David et al. (2023), who identified a notable correlation between the occurrence of an ayahuasca-induced personal death experience and subsequent positive shifts in attitudes towards death. 

Similar links between death attitudinal change and navigating specifically intense and challenging moments of psychedelic experiences, gaining psychological insights, and the occurrence of a mystical-type experience have been noted in various qualitative analyses (e.g. Fisher 1970; Grof et al. 1973; Doblin 1991; Swift et al. 2017; Maia et al. 2021; Michael et al. 2023). The relationship between these acute elements of psychedelic experiences and alterations in death attitudes represents a critical field for further inquiry. For instance, future research may consider delving into the specific facets of the MEQ to establish which aspects of the experience may best predict changes in death anxiety. More broadly, given the correlation between mystical experiences and reductions in death anxiety, identifying which contextual factors—namely set and setting—may facilitate the occurrence of a mystical experience is an additional critical point of inquiry.

Despite the range of methodologies regarding set and setting across the reviewed studies, there was no direct examination of how these factors specifically impact changes in death anxiety. It has been previously established that set and setting are crucial for facilitating mystical-type experiences (Leary 1963; Ko et al. 2022), which, in turn, have been linked to reductions in death anxiety within this review's findings. This suggests an indirect relationship where set and setting may influence death anxiety by fostering conditions conducive to mystical experiences. Investigating how set and setting contribute to the occurrence of mystical experiences might provide deeper insights into optimizing psychedelic-assisted therapies for addressing existential concerns. Nevertheless, the consistent observation of decreased death anxiety across varied research settings — from naturalistic to highly controlled environments — indicates that the mitigation of death anxiety could be an inherent, frequent outcome of psychedelic experiences, regardless of set and setting. An important point of inquiry hereby arises—the connection between psychedelically-induced reductions in death anxiety or improved death attitudes and enduring effects.

Given the significant role of death anxiety in a wide array of mental health conditions (Iverach et al. 2014; Menzies and Menzies 2023), it is conceivable that reductions in death anxiety could underlie some of the therapeutic benefits observed with psychedelic-assisted therapies (Moreton et al. 2020). Nonetheless, the long-term implications of changes in death anxiety following psychedelic experiences remain largely unexplored. While numerous studies have included death anxiety among their variables of interest, detailed analyses of its correlations with long-term outcomes are seldom reported. Only three studies quantitatively assessed the relationship between decreased death anxiety and other enduring outcomes, including sustained improvements in well-being (Moreton, Arena et al. 2023a), as well as reductions in obsessive–compulsive symptoms (Moreton, Burden-Hill et al. 2023b). Additionally, various qualitative accounts suggest that improved death attitudes were connected to improved interpersonal dynamics (Richards et al. 1972; Swift et al. 2017), increased quality of life (Gasser et al. 2015), and an increased sense of meaning in life (Fisher 1970). This gap points to a crucial area for future research, emphasizing the need to systematically investigate the enduring impacts—both positive and negative—of psychedelically induced shifts in death anxiety on broader mental health outcomes.

Our review revealed numerous instances where the reporting of potential adverse effects was ambiguous, highlighting the need for this to become a focal point in future investigations. Specifically, we noted a prevalent lack of detailed measurement and documentation (including in more recent studies) regarding cases of potentially increased death anxiety, with many studies emphasizing average positive outcomes without addressing potential cases of individual detriment. Given that some studies did report instances of heightened death anxiety, it is critical to thoroughly investigate this possible consequence of psychedelic use. Future studies should aim to delve into the phenomenology, frequency, intensity, and causal factors behind instances of exacerbated death anxiety following psychedelic experiences.

### Weaknesses in the measurement of death attitudes, the role of expectation, and blinding efforts

The studies reviewed employed a wide variety of outcome measures, making it challenging to compare results across different research due to the nuanced nature of death attitudes. Particularly, this comparison becomes complex in studies where the study of death attitudes was a peripheral focus, often using single-item measures to assess whether these attitudes have improved or worsened. Given that negative attitudes towards death— especially fear of death — are inherently multifaceted, brief and generalized measures may fail to capture these complexities adequately (Menzies and Menzies 2023). Among the reviewed studies, sixteen included qualitative analyses that reported on changes in death attitudes, yet none deeply explored the phenomenological changes in death anxiety that may occur following psychedelic experiences. A thorough understanding of how psychedelic experiences might alleviate death anxiety is crucial, not just for psychedelic research but also for any potential applications in wider psychotherapeutic practices. This underscores a significant gap in the literature, pointing to the need for detailed qualitative research into the specific nature of the mechanisms whereby psychedelics may influence attitudes towards death.

This review highlighted a significant gap in the research regarding the impact of expectancy and blinding on psychedelic-induced alterations in death attitudes. Given the increasing scrutiny over the potential of expectancy to exaggerate treatment effects, especially in contexts where blinding is challenging (van Elk and Fried 2023), this area requires further investigation (e.g. Szigeti et al. 2024).

The early psychedelic studies included in this review seldom employed blinding techniques, but this practice has increased in more contemporary research. Among the studies reviewed, eight were conducted under double-blind conditions, yet these studies either did not measure or did not report the effectiveness of blinding among participants. The success of blinding efforts for guides, however, was documented in four studies and appeared to be relatively effective in three studies (Griffiths et al. 2011, 2016, 2018) and unsuccessful in one (Ross et al. 2016). The role of expectancy in influencing outcomes of psychedelic therapy remains a critical—yet underexplored question—with no studies in the review assessing the role of expectancy on moderating effects of psychedelic experiences on death attitudes and death anxiety. Future research in the effects of psychedelics and death anxiety may consider the useful practice of measuring expectancy at baseline (as in Szigeti et al. 2024), as well as unique trial designs better equipped to handle unblinding (see Muthukumaraswamy et al. 2021).

### Additional methodological limitations

The review highlighted several critical limitations in the current research on psychedelics and death anxiety. Primarily, a significant degree of selection bias is evident across many studies. This bias is notably present in studies that recruited participants from online forums, where individuals likely to volunteer for scientific research on psychedelics may already hold positive views towards these substances, potentially skewing results away from those of the average psychedelic user. Additionally, certain studies introduced selection bias by recruiting participants based on their reported experiences, such as those who have had “meaningful experiences” or specific changes in death anxiety. These approaches, ranging from indirect to direct selection based on outcome variables, further complicate the generalizability of results. Selection bias is also a concern within clinical trials, which tend to employ strict exclusion criteria, potentially limiting the representativeness of their samples. Moreover, the relatively small sample sizes common in psychedelic research may impact the reliability and robustness of the findings, underscoring the necessity for larger-scale studies to validate these preliminary insights.

An additional limitation must be acknowledged within this review itself; namely, the inclusion of only articles in English. This was due to the concern that efforts made to translate non-English articles may compromise the accuracy of the review due to translation error, particularly considering the complex and nuanced nature of this topic. Death attitudes may vary widely between different cultures (Gire 2014), and it must be acknowledged that this review may have an overrepresentation of Western perspectives.

## Conclusion

The nature of a scoping review does not lend itself to drawing definitive conclusions about the evidence base in the manner of a systematic review or meta-analysis, and the limitations of the current literature further restrict our ability to definitively assess the reliability and impact of psychedelics on death anxiety. However, despite these methodological hurdles, there is a largely consistent narrative across the literature suggesting that psychedelics frequently induce shifts in death attitudes, often towards more positive perceptions. This narrative has threaded through the fabric of psychedelic research from its inception with early explorers like Walter Pahnke and Stanislav Grof to contemporary investigations, highlighting the enduring relevance of this topic. The burgeoning field of psychedelic studies would benefit from methodologically robust research characterized by larger, more diverse sample sizes, stringent controls, and designs that enhance the representativeness of the study populations. Such advancements are crucial for a more accurate understanding of the causal relationships between psychedelic use and changes in death attitudes. Furthermore, there is a clear need for in-depth exploration into the specific mechanisms underlying both the beneficial and adverse shifts in death attitudes, alongside a closer examination of the instances and intensity of increased death anxiety.

## Data Availability

Not applicable.
